# The Effect of Trait Mindfulness on Teachers’ Emotional Exhaustion: The Chain Mediating Role of Psychological Capital and Job Engagement

**DOI:** 10.3390/healthcare9111527

**Published:** 2021-11-09

**Authors:** Yufang Bi, Xindong Ye

**Affiliations:** 1 Psychological Counseling Center, Shanghai University of Engineering Science, Shanghai 200336, China; biyufang2021@163.com; 2 School of Teacher Education, Wenzhou University, Wenzhou 325035, China

**Keywords:** trait mindfulness, emotional exhaustion, psychological capital, work engagement

## Abstract

Emotional exhaustion has become an important occupational health problem faced by teachers, and it has seriously affected their mental health. It is necessary to pay attention to the factors that affect emotional exhaustion. In this study, 815 frontline university faculty were selected as subjects to explore the relationship between trait mindfulness and emotional exhaustion and the role of psychological capital and work engagement in this relation, using the trait mindfulness, psychological capital, work engagement, and emotional exhaustion scales. It was found that trait mindfulness and emotional exhaustion are negatively correlated; the mediating role of psychological capital between trait mindfulness and emotional exhaustion is not significant; the mediating role of work engagement between trait mindfulness and emotional exhaustion is significant; the chain mediation effect of psychological capital and work engagement between trait mindfulness and emotional exhaustion are significant.

## 1. Introduction

With the development of modern society, work pressure is increasing and the emotional exhaustion of teacher groups is also becoming increasingly significant [1], which not only affects the physical and mental health of the teacher groups, but also harms the teachers’ teaching motivation and efficiency [2]. It can also lead to teachers’ failure to properly handle students’ problems [3]. Emotional exhaustion is the core element of job burnout [4,5], which refers to the exhaustion of a person’s emotional resources and is characterized by emotional negativity and lack of energy [6]. It has become a serious occupational health problem [7]. Therefore, it is of great urgency to study the factors that affect emotional exhaustion, so as to provide a reference for helping teachers alleviate emotional exhaustion, improve their physical and mental health, and consequently promote the quality of education and teaching.

The job demands–resources model (JD-R) conceptualizes trait mindfulness as a personal resource, which can affect job requirements and resources, thereby influencing teachers’ emotional exhaustion [8]. Research has shown that mindfulness is negatively correlated with emotional exhaustion [7], and it can effectively relieve teachers’ emotional exhaustion [9]. According to the JD-R model, the characteristics of any job can be divided into job requirements and job resources, and job resources can buffer the negative impact of high job requirements on employees [10]. Emotional exhaustion, as a characteristic of job burnout, is a result of excessive work requirements [11], and therefore will be affected by work resources. Studies have shown that both psychological capital and work engagement belong to work resources [12,13]. Therefore, mindfulness may affect emotional exhaustion by influencing these two kinds of work resources. In summary, this article attempts to explore the influence of mindfulness, psychological capital, and work engagement on university faculty’s emotional exhaustion and the internal mechanism between them, so as to provide an effective way to alleviate teachers’ work burnout.

**Figure 1:**
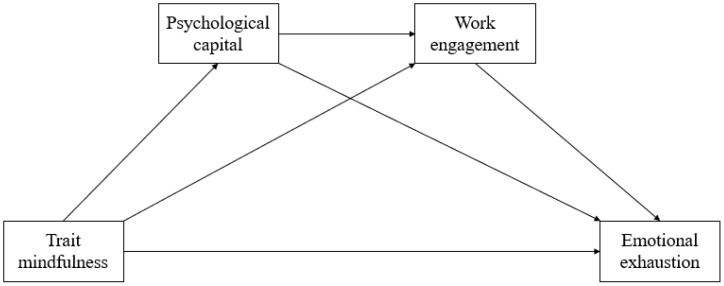
Chain mediating model.

Our research contributes to the emotional exhaustion literature in three ways. Firstly, we explore the relationship between mindfulness and emotional exhaustion. Secondly, we investigate the intermediary mechanism between mindfulness and emotional exhaustion in the view of psychological capital and work engagement. Thirdly, we test the chain mediation effect of psychological capital and work engagement between mindfulness and emotional exhaustion. The specific model is shown in Figure 1.

## 2. Theory and Hypothesis

### 2.1. Trait Mindfulness and Emotional Exhaustion

Mindfulness is derived from the concept and training methods of Buddhist meditation, known as a state of consciousness in which one is fully engaged and self-committed [14]. It consists of three main components: awareness, attention, and memory [15]. Mindfulness can refer either to the psychological traits of mindful awareness as a manifestation of sustained consciousness, or as a state of conscious openness to non-judgmental living [16]. It can be viewed as a behavior (i.e., mindfulness meditation), mental state, psychological process, or even a personality or personality-like trait [17]. Therefore, mindfulness research divides mindfulness into trait mindfulness and state mindfulness [16]. Trait mindfulness refers to the idea that all individuals have different levels of trait mindfulness abilities, while state mindfulness means that mindfulness meditation can cultivate individual mindfulness awareness through basic mindfulness training [18].

With further research on mindfulness, the relationship between mindfulness as an idiosyncratic trait and emotion prediction has begun to draw people’s attention [19]. Studies have shown that trait mindfulness is negatively correlated with perceived stress [20], while emotional exhaustion is just an undesirable consequence of the individual’s perceived stress. Therefore, a high level of trait mindfulness may be related to a low level of emotional exhaustion. Based on the JD-R model, personal resources are seen as the foundation of work needs or resources, while trait mindfulness is a type of personal resources, and it can lower the individual’s assessment of work pressure and enhance their faith of work, thereby affecting teachers’ emotional exhaustion [8]. Therefore, when an individual has a higher trait of mindfulness as a personal resource, he will experience lower emotional exhaustion. Studies have shown that mindfulness can allow individuals to accumulate not only personal resources, but also work resources [8]. Sufficient work resources can alleviate the disadvantages of excessive work requirements [21] such as emotional exhaustion [11]. In summary, mindfulness can reduce emotional exhaustion by helping individuals accumulate more resources. Thus, mindfulness and emotional exhaustion are negatively correlated [22]. Therefore, as a kind of mindfulness, trait mindfulness may also be negatively correlated with individual emotional exhaustion. Hence, we pose Hypothesis 1:

**Hypothesis** **1** **(H1).** *Trait mindfulness is negatively correlated with emotional exhaustion*.

### 2.2. The Mediating Role of Psychological Capital

Psychological capital is the positive mental state shown by the individual in their process of growth and development and is a psychological resource that promotes individual growth and improvement. It mainly includes the four core elements of optimism, hope, resilience, and self-efficacy [23]. Some researchers have found that mindfulness can promote the accumulation of psychological capital [24,25], which can help to improve individual psychological capital [26,27]. Research has also found that trait mindfulness can affect an individual’s psychological capital [28]. As a type of mindfulness, it can positively affect an individual’s psychological capital [29].

According to the JD-R model, work resources are the “positive factor” in work. Work resources can relieve the negative impact of high work requirements on employees [10], such as job burnout. For instance, if an individual’s work resources are abundant, the negative effects brought about by the work are more likely to be lower. Psychological capital is a kind of work resource. Research has also shown that psychological capital is negatively correlated with job burnout [30]. Individuals with high levels of psychological capital may have lower levels of emotional exhaustion [31]. Meanwhile, according to the JD-R model, trait mindfulness can affect the emotional exhaustion of teachers by affecting work requirements and resources [8]. Therefore, psychological capital, as a work resource, can be viewed as a specific pathway through which trait mindfulness impacts emotional exhaustion. In conclusion, trait mindfulness may be able to influence emotional exhaustion through the work resource of psychological capital. Thus, we pose Hypothesis 2:

**Hypothesis** **2** **(H2).** *Psychological capital plays a mediating role in trait mindfulness and emotional exhaustion*.

### 2.3. The Mediating Role of Work Engagement

As a positive work state, work engagement has aroused widespread attention in the past ten years with the rise of positive psychology and positive organizational behavior. Work engagement is a mental state in which positive emotions and motivation related to work are continuously activated, and it performs in three aspects: vitality, dedication, and concentration [32]. This concept was initially intended to describe a persistent and emotional motivational state of employees [33]. Studies have shown that mindfulness is positively correlated with work engagement [34]. It can help individuals make full use of resources, actively participate in work, promote physical and mental health, improve happiness, and thereby increase work engagement [35,36]. Therefore, trait mindfulness may also have a positive relationship with work engagement.

Many studies have shown that there is a negative correlation between work engagement and emotional exhaustion, which means that high-level work participation results in lower presence of emotional exhaustion [37,38]. Some scholars have found that work engagement increases the emotional exhaustion of irresponsible individuals, while it reduces the emotional exhaustion of emotionally stable individuals [39]. According to the JD-R model, abundant work resources make a difference by increasing individual work engagement. That is, work engagement plays an intermediary role [21]. Therefore, trait mindfulness enables employees to make full use of resources, thereby enhancing teachers’ work engagement and assisting teachers to experience lower levels of emotional exhaustion. We conclude that the positive effect of trait mindfulness work on emotional exhaustion may also occur through the pathway of work engagement. Hypothesis 3 is posed as follows:

**Hypothesis** **3** **(H3).** *Work engagement plays a mediating role between trait mindfulness and emotional exhaustion*.

### 2.4. The Chain Mediating Role of Psychological Capital and Work Engagement

The JD-R model believes that personal resources (such as psychological capital) have inherent motivational potential and can lead to higher work engagement [40], while individuals with low psychological capital cannot actively engage in work [41]. Research also shows that psychological capital can promote positive behavior [42]. The hope and optimism in psychological capital can increase the level of work engagement [43]. There is a significant positive correlation between psychological capital and work input [44,45]. Additionally, in the process of some antecedent variables affecting work engagement, psychological capital often plays a mediating role [46,47]. Psychological capital can play a mediating role between mindfulness and work engagement by increasing positive emotions, hope and optimism [36], while mindfulness has a positive impact on work engagement by increasing the hope in psychological capital [44,48]. Therefore, individuals with high-level trait mindfulness may be more abundant in psychological capital, tending to have positive emotions, hope, and optimism to achieve higher work engagement and ultimately experience lower levels of emotional exhaustion. That is to say, the trait mindfulness may enhance the individual’s work input level by increasing the psychological capital and thereby reducing the individual’s emotional exhaustion level.

In summary, we proposed Hypothesis 4:

**Hypothesis** **4** **(H4).** *Psychological capital and work engagement play a chain mediating role between trait mindfulness and emotional exhaustion*.

## 3. Methods

### 3.1. Sample and Procedure

A questionnaire survey was conducted among university faculty selected from three comprehensive public universities. The subjects were told the purpose of the investigation before the survey, and that the data would only be used for scientific research to protect personal privacy. To dispel the subjects’ concerns and ensure data quality, the research process followed ethical requirements, and the subject information was strictly confidential. A total of 830 university faculty participated in the survey, and 820 copies were recovered, with a response rate of 98.79%. After removing the random and missing questionnaires, a total of 815 valid questionnaires were received, with a valid rate of 99.39%. Among them, 633 were women, accounting for 77.7%, and 182 were men, accounting for 22.3%. The age distribution mainly ranged from 26 to 50 years old (85.9%).

This study was approved by the academic committee of the author’s university and complied with the 1964 Declaration of Helsinki. The investigation was conducted with the approval of the school and the teachers themselves, and an informed consent form was signed.

Data were collected on the Wenjuanxing online platform. The collected data includes demographic information (gender, age, education, marital status, working years, etc.), and items on the trait mindfulness, psychological capital, work engagement, and emotional exhaustion questionnaires.

### 3.2. Measures

#### 3.2.1. Trait Mindfulness Scale

Trait mindfulness was measured using the Chinese version of the Mindfulness Attention Awareness Scale (MAAS) developed by Brown and Ryan (2003) [49] and revised and tested by Chen et al. (2012) [50]. The scale is a one-dimensional structure with 15 items in total. Sample items were: “I often don’t realize that I’m in certain emotions, and don’t notice it until sometime later”, “I often break or overturn things due to carelessness, distraction, or thinking about other things”, etc. A 6-point scale system was used for scoring: 1 = always and 6 = never. Cronbach’s α = 0.890. The test–retest reliability was 0.870.

#### 3.2.2. Psychological Capital Scale

This research used the psychological capital scale developed by Luthans et al. and translated by Li (2007) [51], with a total of 24 items, such as “I believe that I can analyze long-term problems and find solutions”, and “When I encounter uncertainty at work I usually look forward to the best results when it comes to issues”. A 6-point scale system is used: 1 represents strongly disagree, and 6 represents strongly agree. Cronbach’s α = 0.94.

#### 3.2.3. Work Engagement Scale

This study uses the Utrecht work engagement scale developed by Schaufeli et al. (2006) [52], with a total of 9 items. Sample items were “At work, I feel myself bursting with energy”, “At work, I feel strong and full of energy.” and so on. A 7-point scale system is used, with 1 representing never and 5 representing always. Cronbach’s α = 0.94.

#### 3.2.4. Emotional Exhaustion Scale

This study uses the emotional exhaustion scale developed by Maslach et al. (1986) [6] and revised by Aryee et al. (2008) [53], with a total of 6 items. Sample items were “My work makes me mentally exhausted”, “I feel exhausted after a day’s work”, and so on. A 5-point scale system is adopted, with 1 representing strongly disagree and 5 representing strongly agree. Cronbach’s α = 0.889.

#### 3.2.5. Control Variables

In line with previous studies, current research takes the teachers’ gender, educational background, marital status, and working years as the control variables.

## 4. Results

### 4.1. Confirmatory Factor Analysis

The Harman single factor test method was performed to test the common method deviation. The explanation rate of the first factor was 35.59%, which was lower than 40%, indicating that there is no serious problem of common method deviation.

### 4.2. Discriminant Validity Test

A confirmatory factor analysis (CFA) was conducted on the four factors of mindfulness, psychological capital, work engagement, and emotional exhaustion to obtain the discriminative validity of the questionnaire (Muthén & Muthén, 2010). The results showed that the four-factor model (χ^2^/df = 2.10, RMSEA = 0.05, CFI = 0.91, TLI = 0.91, SRMR = 0.06) fit the data significantly better than other competitive models; that is, the discrimination validity between the measured variables is acceptable (see Table 1).

### 4.3. Descriptive Statistics and Correlation Analysis

Correlation tests found that trait mindfulness was positively correlated to psychological capital (*r* = 0.573, *p* < 0.01), work engagement (*r* = 0.431, *p* < 0.01), and negatively correlated with emotional exhaustion (*r* = −0.435, *p* < 0.01); psychological capital is positively correlated to work investment (*r* = 0.611, *p* < 0.01) and negatively correlated to emotional exhaustion (*r* = −0.396, *p* < 0.01); work investment is positively correlated to emotional exhaustion (*r* = −0.412, *p* < 0.01), marital status, and working years (see Table 2).

### 4.4. Hypothesis Testing

**Figure 2:**
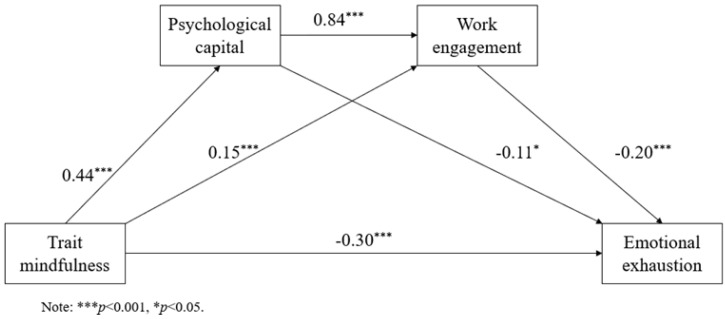
The chain mediating role of psychological capital and work engagement.

The bootstrap method was used to draw 5000 samples to test the mediating effect of psychological capital and work engagement (see Figure 2). The results show that the indirect effect of psychological capital as the mediating variable was −0.05 (95%CI = [−0.11,0.01]), which was not significant; the indirect effect of work engagement as the mediating variable was −0.03 (95%CI = [−0.05,−0.01]), which was significant; the indirect effect of work engagement and psychological capital as the mediating variables was −0.07 (95%CI = [−0.11,−0.04]), indicating the chain mediation effect was significant; the total effect was significant, with an estimated value of −0.15 (95%CI = [−0.21,−0.10]) (see Table 3).

## 5. Discussion

### 5.1. Theoretical Implications

#### 5.1.1. Trait Mindfulness and Emotional Exhaustion

The research shows that there is a negative correlation between trait mindfulness and emotional exhaustion; that is, the higher the level of trait mindfulness, the less easily the emotion is depleted. Thus, Hypothesis 1 is supported.

Teachers will encounter pressure during working because they need to meet the expectations and requirements from students, parents and schools [54], which will cause teachers’ emotional distress [55]. Teachers are expected to show positive emotions to their students in the classroom, which is known as emotional labor [56]. When the emotions expressed by teachers are inconsistent with their actual emotions, emotional exhaustion will occur [57]. The reason why people with higher level of trait mindfulness are less likely to suffer from emotional exhaustion is that they regard bad emotional experiences as simple feelings instead of habitually interpreting them as having a negative impact on themselves [58]. Focusing on this inner experience can free them from negative assessments that lead to emotional exhaustion [59]. This research also further validates the JD-R model, indicating that mindfulness can alleviate individual’s emotional exhaustion. When individuals spend a lot of time, energy, and other resources, they will experience a strong sense of fatigue. Trait mindfulness may help to replenish the resources in time as a personal resource, thereby reducing emotional exhaustion.

#### 5.1.2. The Mediating Role of Psychological Capital

The research suggests that the mediating effect of trait mindfulness through psychological capital is not significant and does not support Hypothesis 2.

This study found that trait mindfulness is positively correlated with psychological capital, which is consistent with previous research results [28]. This may be because increasing individual’s mindfulness level helps to improve their positive psychological characteristics [26]. Research has also shown that mindfulness helps to eliminate negative cognitions by returning individual’s attention to the current task and dealing with their negative cognitions [60].

When an individual’s psychological capital level is high, they can effectively respond to the various pressures they meet and prevent the generation of related negative emotions [61]. Improving individual’s psychological capital can promote their psychological health and relieve negative emotions [62]. However, in this study, the mediating effect of psychological capital is not significant, indicating that there may be other variables accounting for the indirect effects. For example, when an individual puts the existing psychological capital resources outside the workplace, although his psychological capital level is high, the work resources cannot be enriched, and the individual’s emotions may still be consumed. Therefore, future research can further explore the specific factors that have an impact on the role of psychological capital.

#### 5.1.3. The Mediating Role of Work Engagement

It is shown from the study that trait mindfulness affects emotional exhaustion through the mediating effect of work engagement, which supports Hypothesis 3.

Mindfulness is a special internal resource of an individual, which helps to enhance the positive psychological resource of work engagement. Mindfulness is conducive to positive results related to work [29]. Mindful individuals are more autonomic and less defensive to the stimuli at work. This makes them less likely to be affected by the negative work environment and more actively invest in their own work [63]. On the other hand, with the increasing of an individual’s work engagement, the individual’s vitality, dedication and concentration also increases [64]. There is no doubt that the work resources and personal resources are very important to improve our ability [65], which was proven in the work resource research of the JD-R model. Individuals with trait mindfulness will become more focused and engaged in their work than others, which will eventually lead to positive results. With the increase of an individual’s ability, the individual’s passion and enthusiasm for work will also increase [66].

#### 5.1.4. The Chain Mediating Role of Psychological Capital and Work Engagement

The research shows that psychological capital and work engagement play a chain mediating role in the relationship between trait mindfulness and emotional exhaustion. Hypothesis 4 is supported.

This research introduces the role of psychological capital and work engagement in the relationship between trait mindfulness and emotional exhaustion, which is an extension of previous research. Previous studies have shown that mindfulness can buffer individual negative emotions [67]. When possessing the trait of mindfulness, people can be fully aware of their feelings and emotions without judgment. When facing a negative environment or a stressful event, individuals with a high level of mindfulness will be aware of but not stay in an unfavorable environment, which also avoids the generation of negative emotions [68]. Mindfulness can increase an individual’s psychological capital level [69]. When an individual’s psychological capital level is high, he has sufficient resources to cope with work difficulties and increase his work engagement. With the increases of work engagement level, the individual’s competency needs will also increase, thereby buffering the individual’s emotional exhaustion. At the same time, from the role of psychological capital, an individuals’ work attitude and work behavior are an important reflection the role of psychological capital [70]. When the level of trait mindfulness is higher, the individual tends to show a higher level of psychological capital. After the improving of an individual’s psychological capital level, the psychological capital needs to be directed to work resources to have an impact on the individual. On the one hand, it can be reflected by the individual’s work engagement, that is, the psychological capital affected by the trait mindfulness have an influence on the individual’s emotional exhaustion by through work engagement. This also further validates the JD-R model. An individual’s resources are the antecedent variables of work resources [8], and individual’s resources affect the results of work by influencing work resources. Specifically, trait mindfulness, a personal resource, ultimately affects the results of university faculty’s emotional exhaustion by affecting two kinds of work resources: psychological capital and work investment.

In conclusion, the theoretical implications of this study include the following: firstly, this study found a negative correlation between trait mindfulness and emotional exhaustion of university faculty, clarifying the relationship between trait mindfulness and emotional exhaustion. Secondly, the mediating mechanism between trait mindfulness and emotional exhaustion was found to be work engagement rather than psychological capital, which provided a reference for future researchers to study the role of psychological capital. Third, we found a chain mediating effect between trait mindfulness and work engagement, which clarified one of the pathways by which trait mindfulness affects emotional exhaustion.

### 5.2. Practical Implications

This study explored the relationship between trait mindfulness and emotional exhaustion and tested for factors that affect emotional exhaustion. The results show that there is a negative correlation between trait mindfulness and emotional exhaustion. Therefore, school leaders should actively organize mindfulness training. Using formal or informal mindfulness training methods [33], such as mindfulness practice (including full body scan meditation with audio of mindfulness training, muscle relaxation, and abdominal breathing relaxation) to improve university faculty’s mindfulness level and enable them to cope with stressful environments. The research in this article shows that psychological capital and emotional exhaustion are negatively correlated. In addition to paying attention to the education and training of students, schools should also improve the psychological capital level of university faculty by giving positive feedback, setting oriented goals, and providing organizational support. Finally, there is a negative correlation between work engagement and emotional exhaustion, which indicates that colleges and universities should improve the working environment and work treatment, and actively organize activities conducive to university faculty’s physical and mental health, so as to improve the enthusiasm of college teachers and increase their work involvement. In addition, because university faculty have both teaching and scientific research work, the dual job requirements will affect the level of university faculty’s work involvement. Therefore, university faculty should arrange their scientific research time reasonably, and universities should also arrange the teaching time of college teachers reasonably and put forward reasonable scientific research requirements.

## 6. Conclusions

This study shows that the higher the university faculty’s trait mindfulness level is, the less often emotional exhaustion occurs; the trait mindfulness can affect the university faculty’s emotional exhaustion through work engagement; the trait mindfulness can also affect the psychological capital and then the work engagement, and ultimately affect the university faculty’s emotional exhaustion.

## Figures and Tables

**Table 1:** Results of confirmatory factor analysis of the measurement models (*N* = 815).

Model	χ^2^/*df*	RMSEA	CFI	TLI	SRMR
Four-factor a	2.1	0.05	0.91	0.91	0.06
Three-factor b	7.45	0.09	0.71	0.71	0.09
Two-factor c	9.74	0.1	0.62	0.6	0.1
One-factor d	11.28	0.11	0.55	0.53	0.1

Note: a, hypothesis model; b trait mindfulness, psychological capital combined into one factor; c trait mindfulness, psychological capital and work engagement combined into one factor; d combined into one factor.

**Table 2:** Descriptive statistics and correlations among study variables (*N* = 815).

Variables	*M*	*SD*	1	2	3	4
Gender	-	-				
Educational background	-	-				
Marital status	-	-				
Working years	-	-				
Trait mindfulness Psychological capital Work engagement Emotional exhaustion	4.346	0.799	-			
	4.322	0.632	0.573 **	-		
	4.545	0.974	0.431 **	0.611 **	-	
	2.932	0.822	−0.435 **	−0.396 **	−0.412 **	-

Note: ** *p* < 0.01. Gender: 1 = male, 2 = female; Educational background: 1 = junior college degree or below, 2 = bachelor’s degree, 3 = master’s degree, 4 = doctoral degree or above; Marital status:1= unmarried, 2 = married, 3 = divorced; Working years: 1 = 1–5 years, 2 = 6–10 years, 3 = 11–15 years, 4 = 16–20 years, 5 = 21–25 years, 6 = 26–30 years, 7= more than 30 years.

**Table 3:** The chain mediating role of psychological capital and work engagement.

Effect Category	Effect Value	SE	BootLLCI	BootULCI
Total indirect effect	−0.15	0.03	−0.21	−0.10
Indirect effect1: trait mindfulness- > psychological capital- > emotional exhaustion	−0.05	0.03	−0.11	0.01
Indirect effect2: trait mindfulness- > work engagement- > emotional exhaustion	−0.03	0.01	−0.05	−0.01
Indirect effect3: trait mindfulness- > psychological capital- > work engagement- > emotional exhaustion	−0.07	0.16	−0.11	−0.04
Indirect effect1-Indirect effect2	−0.02	0.03	−0.09	0.04
Indirect effect1-Indirect effect3	−0.02	0.04	−0.05	0.10
Indirect effect2-Indirect effect3	−0.04	0.02	−0.01	0.08

## Data Availability

The data shall be obtained with the consent of the corresponding author.
